# Optimising quality of life for people living with heart failure in care homes: Protocol for the co-design and feasibility testing of a digital intervention

**DOI:** 10.1371/journal.pone.0288433

**Published:** 2023-07-11

**Authors:** James McMahon, Christine Brown Wilson, Loreena Hill, Paul Tierney, David R. Thompson, Jan Cameron, Doris Yu, Debra K. Moser, Karen Spilsbury, Nittaya Srisuk, Jos M. G. A. Schols, Mariëlle van der Velden, Gary Mitchell

**Affiliations:** 1 School of Nursing and Midwifery, Queen’s University Belfast, Belfast, United Kingdom; 2 School of Clinical Sciences at Monash Health, Monash University, Melbourne, Australia; 3 School of Nursing, University of Hong Kong, Hong Kong, China; 4 College of Nursing, University of Kentucky, Lexington, Kentucky, United States of America; 5 School of Healthcare, University of Leeds, Leeds, United Kingdom; 6 Faculty of Nursing, Surat Thani Rajabhat University, Surat Thani, Thailand; 7 Department of Health Services Research and Department of Family Medicine, Care and Public Health Research Institute (CAPHRI), Maastricht University, Maastricht, The Netherlands; Swiss Paraplegic Research, SWITZERLAND

## Abstract

**Background:**

Heart failure (HF) affects up to 64.3 million people globally. Advancements in pharmaceutical, device or surgical therapies, have led to patients living longer with HF. Heart failure affects 20% of care home residents, with these individuals presenting as older, frailer, and with more complex needs compared to those living at home. Thus, improving care home staff (e.g., registered nurse and care assistant) knowledge of HF has the potential to benefit patient care and reduce acute care utilization. Our aim is to co-design, and feasibility test, a digital intervention to improve care home staff knowledge of HF and optimise quality of life for those living with the condition in long-term residential care.

**Methods:**

Using a logic model, three workstreams have been identified. Workstream 1 (WS1), comprised of three steps, will inform the ‘inputs’ of the model. First, qualitative interviews (n = 20) will be conducted with care home staff to identify facilitators and barriers in the provision of care to people with HF. Concurrently, a scoping review will be undertaken to synthesise current evidence of HF interventions within care homes. The last step will involve a Delphi study with 50–70 key stakeholders (for example care home staff, people with HF and their family and friends) to determine key education priorities related to HF. Using data from WS1, a digital intervention to improve care home staff knowledge and self-efficacy of HF will be co-designed in workstream 2 (WS2) alongside those living with HF or their carers, HF professionals, and care home staff. Lastly, workstream 3 (WS3) will involve mixed-methods feasibility testing of the digital intervention. Outcomes include staff knowledge on HF and self-efficacy in caring for HF residents, intervention usability, perceived benefits of the digital intervention on quality of life for care home residents, and care staff experience of implementing the intervention.

**Discussion:**

As HF affects many care home residents, it is vital that care home staff are equipped to support people living with HF in these settings. With limited interventional research in this area, it is envisaged that the resulting digital intervention will have relevance for HF resident care both nationally and internationally.

## Introduction

The global prevalence of heart failure (HF) is estimated at 64.3 million cases [1]. Due to a growing population, increasing numbers of older people, and improved rates of survival following a cardiovascular event, HF rates are predicted to continue to rise [1, 2]. The mean age of HF patients is 77 years, and they often present with up to five comorbidities that can include chronic kidney disease, chronic obstructive pulmonary disease, and arthritis [3]. An estimated 20,000 people are currently living with HF in Northern Ireland (NI) [4]. Advancements in pharmaceutical, device or surgical therapies, have resulted in patients living longer with HF, thus placing an unprecedented demand on hospital facilities, personnel resources, and incurring considerable health care costs [3, 5]. A retrospective review of nurse-led HF services, across the island of Ireland in 2020, reported a 19% increase in patient consultations compared to 2019, with 900 patients reviewed either virtually or face-to-face [6]. For many older people, the fear of contracting COVID-19, or unfamiliarity with technology required for remote monitoring, led to significant delays in seeking medical help and resulted in irreversible outcomes. Evidence from across Europe demonstrates increasing complexity, with many more people with HF being admitted to hospital during the COVID-19 pandemic [7, 8].

Across the United Kingdom (UK), approximately 400,000 older people live in 17,079 care homes, including both residential homes (providing accommodation and personal care) and nursing homes (providing nursing care) [9]. Heart failure affects 20% of care home residents, causing high rates of morbidity and mortality [10]. An observational study of 15,549 HF patients conducted in North America reported that those recently discharged from hospital to care homes were older, frailer, and with more complex needs, compared to patients discharged to home. As a result, patients discharged to care homes had a higher risk of death and re-hospitalization [11]. Several factors may contribute to this, including insufficient drug prescribing (e.g., Beta-blockers), inadequate monitoring of HF symptoms and renal function for patients on loop diuretics, and limited clinical and guideline-based knowledge of nursing professionals and healthcare assistants [12]. Improving nursing and care assistant knowledge has the potential to benefit patient care, professionals’ job satisfaction, and reduce acute care utilization [13].

Recent qualitative findings from care home staff have identified key themes on which they would like additional learning related to HF. These included knowledge and skills about identification of HF patients, HF signs and symptoms, purpose of daily weighing, indicators of worsening HF, purpose of sodium restricted diet, and materials to improve patients’ understanding of HF [14]. These findings illustrate that it is vital that the care home staff caring for people living with HF are knowledgeable and competent in skills to better support older people living in care homes to manage their condition and maximise their quality of life.

The aim of this study is to co-design a digital intervention to improve care home staff knowledge and self-efficacy of caring for residents with HF, subsequently optimising quality of life for people living with the condition in care homes. Moreover, this study will involve:

- A scoping review to synthesise current evidence on HF interventions within care home settings.
- Qualitative interviews with care home staff to identify facilitators and barriers in the provision of care to people living with HF in care homes.
- A series of questionnaires (Delphi study) to determine what key stakeholders perceive to be the key education priorities related to HF.
- Co-design of a digital intervention with HF residents, their family members, HF professionals from medicine, nursing, pharmacy and other relevant disciplines, and care home staff.
- Feasibility testing of the digital intervention to ass intervention usability, perceived benefits of the digital intervention on quality of life for care home residents, care staff experience of implementing the intervention, and preliminary efficacy of the intervention for improving staff knowledge of HF and self-efficacy in caring for HF residents.

## Methods

### Design

A logic model was used to guide the design of this study, helping to improve the planning of the research study through highlighting theoretical and practical gaps, and guide decisions regarding the methods for data collection and analysis [15]. This study will take two years to complete through three key workstreams:

1. Generating theory
2. Co-design of the digital intervention
3. Feasibility testing

### Workstream 1: Generating theory

Workstream 1 (WS1) will inform the ‘inputs’ of the logic model and will be achieved through three phases. The first two phases will be conducted concurrently, with phase 3 conducted following their conclusion. It is anticipated that WS1 will be completed in the first 12 months of the two-year study. The aim of WS1 is to obtain evidence at a global (scoping review), national (Delphi study), and local (qualitative interviews) scale to inform the design of the digital intervention.

### WS1 phase 1: Scoping review

Phase 1 will involve a scoping review of the literature to synthesise the current evidence on interventions within care homes for improving care of residents living with HF. This will be achieved through three objectives:

- What interventions, if any, have been implemented to improve the provision of care for HF care home residents?
- Have interventions implemented in care homes proven effective in improving HF resident care?
- What are the reported experiences of providing interventions for improving care of HF residents in care homes by care home staff, including challenges, facilitators, and barriers?

The findings of this review will help inform both WS2 and WS3. This review will be conducted in accordance with the Preferred Reporting Items for Systematic reviews and Meta-analyses extension for Scoping Reviews (PRISMA-ScR) checklist [16]. A search of the following databases will be conducted: MEDLINE, CINAHL, Web of Science, EMBASE, WHO International Clinical Trials Registry Platform, UK Clinical Trials Gateway and ClinicalTrials.gov. Example search terms will include nurses, nurse practitioners, heart failure, nursing homes, and residential homes. Reference lists of relevant systematic reviews and scoping reviews will be searched manually to identify additional studies not found in the initial search. Eligible studies will be those comprised of care home residents with HF and/or care home staff involved in providing care to those with HF. Interventions can be delivered through any approach (e.g., in-person or digitally) and must report on outcomes clearly associated with changes to quality of care (e.g., HF knowledge of care home staff and quality of life of residents). Studies reporting only on outcomes associated with HF management (e.g., drug prescribing and monitoring of HF symptoms) will be excluded. Non-interventional studies, those with no clear association to HF, and those that are not implemented exclusively in the care home setting, will also be excluded. A summary of the scoping review protocol is available via the Open Science Framework (registration DOI: https://doi.org/10.17605/OSF.IO/QVXRP).

### WS1 phase 2: Qualitative interviews with care home staff

Semi-structured one-to-one qualitative interviews will be conducted with care home staff from nursing and residential homes to identify facilitators and barriers in the provision of care to people with HF. Interviews will be conducted online using the Microsoft Teams platform. The aim of the one-to-one interviews is to gain an in-depth understanding of the phenomena and inform the co-design of the digital intervention (WS2). In line with recommendations for sample sizes in qualitative research [17], it is anticipated that 20 participants will be recruited, with interviews ceasing once data saturation is achieved. Participants will be recruited through convenience sampling using Queen’s University Belfast (QUB) Northern Ireland Care Home Research Network. This network is comprised of more than 400 individuals working within the care home sector in NI. As part of the network, all members have consented to being contacted by university staff about opportunities to be involved in research, quality improvement or education initiatives. Participants for this study will be recruited through email. The level of experience possessed by participants is likely to vary, with eligible participants practicing in one of the following roles: home manager, charge nurse, nurse, senior care assistant and care assistant. Participants must be currently practicing within the care home setting for more than six months to be considered for inclusion.

### WS1 phase 3: Delphi study

Guided by the findings from the scoping review and one-to-one interviews, a Delphi study will be conducted to determine what key stakeholders perceive to be the key education priorities related to HF. Initial Delphi items will be comprised by the research team based on empirical findings from phase 1 and phase 2 of WS1. Delphi studies present an opportunity for experts to provide anonymous and honest feedback on the area of interest, with the multiple rounds of surveys associated with the technique allowing for reflection and more considered answers [18]. It is anticipated that three rounds of surveys will be undertaken with key stakeholders across the UK (England, Scotland, Wales, and Northern Ireland) for the completion of the Delphi study. Stakeholders will include care home staff, HF practitioners, people living with HF, carers of people living with HF, educationalists, and key charity representatives. Recruitment will be supported by the Royal College of Nursing’s Older People Forum, a UK-wide forum serving approximately 12,000 care staff who provide care to older people. Additional recruitment will be carried out using the research team’s professional networks. It is anticipated that between 50–70 key stakeholders will be recruited to take part in the Delphi study. Surveys will be developed online using MS Forms and emailed to participants that have provided informed consent. Following the final round of survey, a consensus meeting will be held online with key stakeholders to provide them with the opportunity to vote anonymously on the outcomes they consider as key educational priorities that should inform co-design of the digital intervention (WS2).

### Workstream 2: Co-design of the digital intervention

Guided by the findings from WS1, workstream 2 (WS2) will focus on the co-design of the digital intervention. Co-design involves an ongoing collaboration between the research team, developers, and end-users [19]. Collaborating with end-users ensures that their technological and human needs are met, subsequently improving the acceptability and success of the intervention [20]. The digital intervention will be the ‘output’ of the logic model.

Guided by the findings from WS1, the digital intervention will be developed alongside a co-design group. It is envisaged that the co-design group will be comprised of approximately 20 individuals (approximately five people with HF or caring for someone with HF, five healthcare professionals with heart failure expertise, five care home staff and five members of the research team who have clinical/academic experience in HF and care homes). Recruitment to the co-design group will be conducted with assistance from the research team who collectively represent individuals from each group. As the digital intervention will be developed in collaboration with an external technology partner, a nominated person from this organisation will be invited to attend all co-design group meetings. The external technology partner will actively participate in co-design activities by providing input based on discussions in real time and produce prototypes/templates for the co-design team to feedback on at relevant stages during the development process.

It is envisaged that the co-design group will meet once per month over a four-month period during the development process. The first meeting will aim to welcome everyone to the co-design group, gather expert experiences of the topic, and provide the research team with an opportunity to share the findings from WS1. The second meeting will focus on discussing the key priorities of the intervention to highlight what the resource should do. The third meeting will focus on intervention content such as scientific messages and ensuring the language used is appropriate for the end-users. Lastly, the fourth meeting will focus on the design of the intervention, such as layout, navigation, and colours used throughout. These meetings are crucial in ensuring that the intervention meets the needs of the end-users. It is anticipated that all co-design meetings will be conducted face-to face. However, should COVID-19 related restrictions dictate, these co-design meetings can be hosted remotely using Microsoft Teams. This approach to co-design methodology is reflective of the international evidence-base [21–23].

### Workstream 3: Feasibility testing

Applying a mixed method design, workstream 3 (WS3) will produce the ‘outcomes’ of the logic model through feasibility testing of the digital intervention. It is anticipated that WS3 will be achieved during the final 9-months of the project. Underpinned by recommendations for conducting feasibility studies [24], the following areas will be assessed across three phases of data collection:

- Acceptability:
-   *to what extent the intervention is judged as suitable*, *satisfying*, *or attractive to care home staff*
- Demand
-   *to what extent the intervention is likely to be used by care home staff*
- Implementation
-   *to what extent the intervention can be successfully delivered to care home staff*
- Practicality
-   *to what extent can the intervention be carried out with care home staff using existing means*, *resources*, *and circumstances*
- Integration
-   *to what extent the intervention can be integrated into an existing system i*.*e*., *daily practice*
- Limited efficacy
-   *Does the intervention show promise of being successful*

### WS3 phase 1: Care home staff knowledge, self-efficacy, and usability

Phase 1 will involve the assessment of care home staff knowledge and self-efficacy of providing care to HF residents following the implementation of the digital intervention through a pre- and post-test questionnaire. The questionnaire will be administered electronically via the digital intervention. Two validated questionnaires, The Dutch Heart Failure Knowledge Scale (15-item) [25] and the Caregiver Contribution to Self-care of Heart Failure Index (22-item) [26], will be used to assess staff knowledge on HF and self-efficacy in providing care to residents with HF, respectively. Questionnaires will be provided to participants pre- and 3-months post-intervention. Approximately 100 participants will be recruited to complete the questionnaires across five nursing homes and five residential care homes. These participants will be recruited via the Queen’s University Belfast Care Home Research Network as described earlier in the protocol. The ten participating care homes will represent the six counties of Northern Ireland and be situated in both rural and urban areas. Intervention analytics, including user engagement and time spent on the resource, will also be analysed to help determine intervention usability and acceptability. Overall, these outcome measures will indicate the extent to which the digital intervention improved knowledge and impacted on care home staff belief that they are able to provide effective care to people with HF, answering part of the ‘implementation’ and ‘limited efficacy’ component [24]. Intervention usability will be assessed through the validated 10-item System Usability Scale (SUS) questionnaire [27], employed post-test. This test will achieve part of the ‘practicality’ component of the feasibility test [24].

### WS3 phase 2: Residents’ care needs, and quality of life and care

Phase 2 of the feasibility testing, conducted concurrently with phase 1, will aim to identify the care needs of HF residents and whether the digital intervention produced short-term benefits for both quality of care and quality of life. Data collection will be conducted three months post-intervention through one-to-one qualitative interviews with HF residents (and family members if requested by the resident) who live in care homes that received access to the intervention. An appropriate interview guide for this phase will be developed by our co-design team during WS2. Ten residents (one from each participating care home) will be recruited for this phase. In addition, care home managers from the ten sites will be asked to conduct a baseline audit at three months pre-intervention and three months post-intervention to determine if the intervention affected the number of major adverse incidents in residents with HF (for example death or hospitalisation) and quality improvements (for example medication review, development of advance care plan or referral to specialist). The specific details of this audit will be co-designed during WS2. Phase 2 will therefore assess three components of the feasibility test: i) ‘demand’, ii) ‘acceptability’, and iii) limited feasibility [24].

### WS3 phase 3: Staff experience on implementation

The final phase of the feasibility test will occur following the completion of the previous phases. The aim of phase 3 is to understand care home staff experiences of implementing the intervention in their care home. Data gathered during this phase will help inform guidelines for the future implementation of the digital intervention. Focus groups (n = 4) will be conducted with up to 32 care home staff, who experienced using the intervention. It is anticipated that participants will be comprised of home managers, nurses, and non-nurses. Three focus groups will be comprised evenly of frontline care home staff (e.g., nurses, and care assistants) while one focus group will be conducted solely with home managers. Dependant on data saturation, the number of participants may be either increased or decreased. Recruitment will take place following the completion of the post-questionnaires in phase 1, with all participants offered the opportunity to participate in the focus group sessions. If interested, the participant will be asked to contact a member of the research team. Phase 3 will help answer three components related to the feasibility test: i) acceptability, ii) implementation, and iii) integration [24]. A summary of the methods involved in each phase of the feasibility test, along with the associated feasibility components being assessed, can be found in Table 1.

**Table 1:** Phases, methods, and feasibility components.

Phase	Methods	Feasibility component
**Phase 1**	Change in care home staff (n = 100) knowledge and self-efficacy pre- and post-intervention assessed through two questionnaires: I. The Dutch Heart Failure Knowledge Scale (15-item) II. The Caregiver Contribution to Self-care of Heart Failure Index (22-item) Intervention usability assessed post-intervention through the 10-item System Usability Scale (SUS) questionnaire	Implementation Limited efficacy Practicality
**Phase 2**	Qualitative one-to-one interviews with HF residents (n = 10) to explore the short-term benefits that residents living with HF experienced. Conducted three months post-intervention. Pre/Post-Audit on HF Residents three months before and three months after the implementation of the intervention.	Demand Acceptability Limited efficacy
**Phase 3**	Focus groups (n = 4) with care home staff (n<32) to understand the experience of implementing the intervention in their care home	Implementation Integration Acceptability

### Data analysis

#### Qualitative analysis

The one-to-one interviews (WS1), conducted using Microsoft Teams, will be video and audio recorded. It is anticipated that the focus groups (WS3) will be conducted face-to-face, and audio recorded. Recordings for both WS1 and WS2 will be transcribed verbatim and uploaded for analysis to NVivo 11 management software. All qualitative data will be analysed using a thematic analysis framework [28]. One-to-one qualitative interviews will be conducted by one member of the research team, whilst focus groups will be facilitated by two. All members of the research team involved in data collection will have prior experience using the methods chosen.

### Quantitative analysis

Quantitative data analysis will be conducted using SPSS v.26. Descriptive statistics will be employed to report on results of the Delphi study and internal analytics of the digital intervention obtained during the feasibility test e.g., time spent on the intervention by care staff. Dependent t-tests will be used for measuring mean change in care home staff knowledge and self-efficacy post intervention to assess preliminary efficacy. As a feasibility study, thus not statistically powered, results will be interpreted with caution.

### Consent processes

All care home staff, residents, and family members, will provide informed consent to take part in the study. All care home residents involved must have capacity to provide informed consent, and therefore, those living with advanced dementia will not be eligible for face-to-face interviews. The research team will acquire written consent by email or mail. If unable to provide written consent due to physical restrictions, verbal consent will be documented through audio recording. Those who expresses an interest in the study will be provided with a participant information sheet and given the opportunity to discuss the research or ask questions. Those that are interested and meet the inclusion criteria will be formally recruited to take part in the study. Those living with dementia will be identified following discussions with care home staff. Participants will have the right to withdraw from the study at any time.

### Ethics and governance

This study has received ethical approval from the Faculty of Medicine and Health Sciences, Queen’s University Belfast (MHLS22_112 and MHLS23_33). Local governance arrangements will be managed by each individual care home or care home group as these organisations are independent from the National Health Service (NHS) in Northern Ireland. In relation to data management, Microsoft Teams will be used, fully compliant with the Data Protection Act (2018) and has the facility to record meetings to aid note taking and capturing all participants’ views. Video recordings will be downloaded to the secure network in Queen’s University Belfast. Once transcribed, all recordings will be deleted from the secure network. Informed consent will be collected from all participants involved throughout the study, with involvement kept confidential. Participant information will only be available to members of the research team. The identity of participants will be anonymised for all related publications and study outputs.

### Dissemination of findings

It is envisaged that study outputs will be disseminated widely through several methods, including peer-reviewed journal articles, scientific conference presentations and webinars, and on social media platforms (e.g., Twitter and Facebook).

## Discussion

To our knowledge, there is a dearth of research on the development and testing of digital interventions to improve care home staff understanding of HF management and symptom monitoring. A systematic review of 63 interventional studies aimed at changing care staff practices in nursing homes reported that, although achieving change is possible, the process is complex [29]. The authors recommend uncovering barriers and facilitators towards intervention engagement, and to conduct process evaluations with end-users to better understand how and why the intervention was either a success, or failure. By engaging with end-users through one-to-one interviews, a Delphi study, a co-design group, and focus groups, this study will address such recommendations.

It is anticipated that the intervention developed during this study will not only impact care provision for HF residents in in the recruited care homes, but if successful, could also have scalability at both a national and international level in the future. To facilitate impact at an international level, an International Observer Panel (IOP) has been established with seven experts in HF, care of older people or in care home settings (JC, DY, DM, KS, NS, JS, MV). Members of the IOP provided guidance and recommendations in the development of this protocol. The IOP will also monitor project activities throughout and provide expert input into each stage of the study. The IOP will also be able to provide feedback about how the intervention could work or might be adapted in their home countries and support international dissemination activities. It is anticipated that members of the IOP will meet with the research team twice per year using Microsoft Teams. Additional email contact will be held with members of the IOP throughout each stage of the study. Members of the IOP will provide expert feedback and consultation on the project and as such, will be acknowledged as co-authors on all outputs associated with the project.

### Limitations and challenges

Historically, engaging individuals in care homes can prove challenging due to a lack of research knowledge, such as informed consent and time commitments related to participation, acting as a barrier to engaging individuals in a care home setting [30]. However, an indirect impact of the COVID-19 pandemic is an improvement in public health awareness of research [31, 32]. While more research is needed to understand the relationship between an improved awareness of research and engaging individuals in care homes, the recent findings may prove favourable for this study. High rates of staff turnover are well-documented in UK care homes [13], potentially presenting as a challenge through impacting participant retention. Lastly, several challenges have been reported in the implementation of digital technologies within the care home setting. Such challenges include time constraints for care home staff, and that many technologies require assistance from more than one person to work as intended [33–36]. However, through collecting evidence at a global, national, and local scale, and engaging with end-users through co-design, this study will develop an appropriate and well-accepted digital intervention to improve care home staff knowledge of HF.
